# Cross-cultural adaptation and validation of the 2021 Louisiana Needs Assessment Questionnaire for Arabic-speaking people living with HIV

**DOI:** 10.1186/s42506-024-00171-x

**Published:** 2024-10-07

**Authors:** Eman Anwar Sultan, Heba Mahmoud Elweshahi, Fatma Tharwat Mohamed, Mona Ashry

**Affiliations:** 1 Community Medicine Department, Faculty of Medicine, Alexandria University, Alexandria, Egypt; 2 Alexandria Hepatology, Gastroenterology and Fever Hospital, Alexandria, Egypt; 3 College of Medicine, Arab Academy for Science, Technology & Maritime Transport, Alamein, Egypt

**Keywords:** People living with HIV (PLWH), HIV, Needs assessment, Arabic translation, Questionnaire, Cognitive interview, Egypt

## Abstract

**Background:**

Despite the global decline in HIV infections and mortality worldwide, the HIV epidemic is still growing in the MENA region. In the region, People Living with HIV (PLWH) are facing many challenges related to cultural values, norms, and provided services which create significant obstacles to HIV prevention and control efforts. This study aimed to translate, culturally adapt, and validate the “2021 Louisiana Needs Assessment Questionnaire” for use among Egyptians and Arabic-speaking population.

**Methods:**

Arabic translation and cultural adaptation of the questionnaire passed through five stages. The questionnaire was translated forward and backward then an expert committee reviewed the translated version. Another expert committee reviewed the developed version after modification to assess the content validity using the Content Validity Index (CVI). The last step included a cognitive interview of a convenient sample of 50 adult PLWH in five consecutive rounds to assess subjects’ understanding of questions and response items and their meanings.

**Results:**

Modifications were carried out all through the translation and adaptation process of the questionnaire including used words, nomenclature of services, adding or omitting response items, and ordering of questions and response items. The synthesized Arabic-adapted questionnaire has adequate content validity and all questions are clearly understood by the studied subjects. The calculated Content Validity Index of all questionnaire items ranged from 0.82 to 1.

**Conclusion:**

The developed culturally adapted questionnaire has adequate content validity/semantic appropriateness. It can be used to assess the needs of PLWH in the MENA region with minor adaptations to fit each country. It can also be used to follow the outcome and impact of implemented programs and services. Further research is recommended to assess its psychometric properties.

**Supplementary Information:**

The online version contains supplementary material available at 10.1186/s42506-024-00171-x.

## Introduction

Despite the global decline of the number of human immunodeficiency virus (HIV) infections and acquired immunodeficiency syndrome (AIDS) related mortality by 16% and 33%, respectively since 2010, the HIV epidemic is still growing in the Middle East and North Africa (MENA) region. The Joint United Nations Program on HIV/AIDS (UNAIDS) reported a 10% increase in new infections and a 9% increase in mortality between 2010 and 2018. This corresponds to 20,000 new HIV infections in the region in 2018 [1, 2].

It is noteworthy that HIV prevalence remained low among the general population in most of the Region’s countries including Egypt [2]. This is probably due to widely accepted cultural values, norms, and practices, such as male circumcision, a ban on extramarital sex, and alcohol abstinence. Yet, cultural norms also frequently lead to stigma and discrimination against people living with HIV (PLWH) creating significant obstacles to reaching them with HIV prevention, testing, care, and treatment [3, 4].

Anti-retroviral therapy (ART) has significantly altered the course of the disease and reduced the morbidity and mortality associated with HIV. Nevertheless, PLWH still faces many challenges related to comorbidities and adverse consequences of continued therapy. These challenges, in addition to the major psychological problems they are prone to, greatly affect the healthcare needs and services of PLWH [5].

The need to translate questionnaires developed in the English language for use in non-English speaking countries has grown rapidly to reach equivalence between the original source and target versions of the questionnaire. Linguistic translation of items is not enough. “Cross-cultural adaptation” is the process of preparing a questionnaire for use in other settings. It is needed to ensure the validity of the tool across different cultures [6].

A qualitative technique designed to examine whether a survey question achieves its intended goal is the cognitive interview. The purpose of cognitive interviews is to ascertain that the respondents can understand the items of the questionnaire and decide if any modification is needed [7].

The burden and challenges faced by PLWH in Egypt have urged the researchers to investigate the key barriers they confront and the priorities they need for their own health and wellness. This information will be useful for planning and decision-making for HIV programs and services. The first step was to adapt a valid tool to be used for the needs assessment of PLWH in Egypt. Most of the questionnaires are developed in English [8] and to date, there is no valid Arabic questionnaire available to investigate the health needs of PLWH. Therefore, the current work aimed to translate, culturally adapt, and validate the “2021 Louisiana Needs Assessment instrument” [9] to be used among the Egyptian and Arabic-speaking populations.

## Methods

### The 2021 Louisiana Needs Assessment Questionnaire

The 2021 Louisiana Needs Assessment Questionnaire [9] was jointly developed by the Policy and Research Group (PRG) and the Louisiana Department of Health’s Office of Public Health STD/HIV/Hepatitis Program (OPH SHHP). The questionnaire was used to assess PLWH needs including identification of services available, services needed and healthcare challenges facing them aiming to improve access to healthcare services by PLWH.

The original questionnaire is composed of six sections. The first section was designed to collect data regarding health, medical care, and treatment history. The second section was to explore the services needed by PLWH namely core medical services, supportive, and housing services. The third section titled Medical Costs and Health Insurance Coverage aimed to identify the medical costs and how PLWH afford them. The fourth section asked about the HIV medications received. The fifth section was designed to assess the current and past housing situations of PLWH. The sixth section included general information about the study subject namely the sociodemographic data, how the subject gets information about HIV, how many years passed since diagnosis, and the place of diagnosis. The last part included questions about employment and income [9].

### Cross-cultural adaptation: (Fig. 1)

**Fig. 1:**
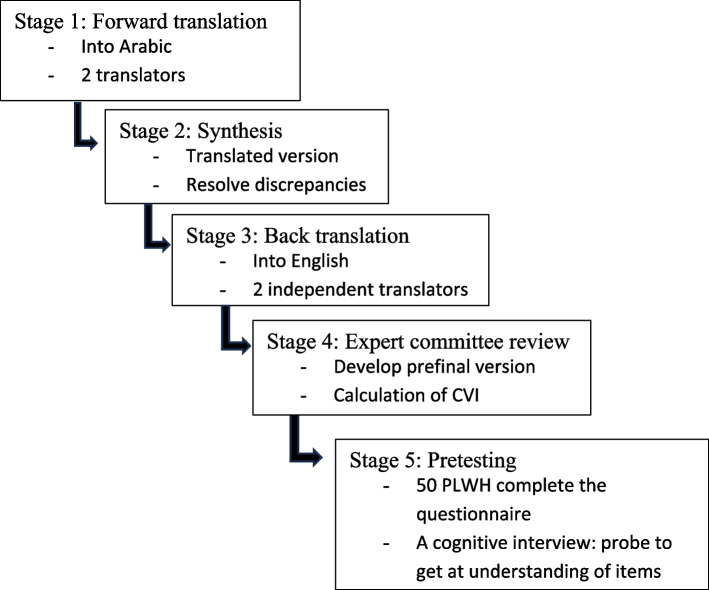
Flow chart of the process of Arabic translation and cultural adaptation of the questionnaire

Arabic translation and cultural adaptation of the questionnaire followed the published guidelines for the process of cross-cultural adaptation of self-report measures [6]. It passed through five stages represented as follows:

1. Forward translation: forward translation of the English version to Arabic was carried out by the third and fourth authors separately. Two translated versions were developed at that stage.
2. Synthesis of a translated version: the two translators compared the developed two versions, discussed and resolved disagreements found, and developed a single agreed-upon translated version.
3. Back translation: the last version was back-translated to the original English language by two independent translators from another medical specialty who were not aware of the intended measures of the questionnaire aiming to find unexpected meanings or misleading words in the translated questionnaire.
4. Expert committee review:
  4.1 The expert committee included, besides the authors, three experts in the field. One expert held a Doctor of Philosophy (PhD) degree with more than 20 years of working experience in the field of HIV and public health. The second expert held PhD degree with more than ten years of working experience in the Egyptian National AIDS Program. The third expert had more than ten years of working experience in Non-Governmental Organizations (NGOs) supporting PLWH. The role of the committee was to verify the translated version and to assess the cultural adaptation of the questionnaire. All translated versions together with the original questionnaire were independently reviewed. They focused in their review on:
    - Cultural adaptation of questions and response items to fit the culture and context of the Arab countries in general and Egypt in particular taking into consideration the nomenclature of services and settings and equivalence of terms.
    - Organization and ordering of sections and questions were reviewed and adapted to the Egyptian and Arabic context.
      The suggested modifications were discussed to reach a consensus about the necessary modifications
  4.2 After applying the final modifications, the researchers presented the translated version to six experts (not those included in the first review). They were asked to rate each item as well as each section of the questionnaire regarding their relevance and clearance. The content validity index (CVI) was calculated for each item to support the validity of the adapted questionnaire [10].

5. Pretesting of the pre-final version: a cross-sectional survey was conducted among 50 PLWH where participants completed the questionnaire and underwent a cognitive interview to assess what they thought about the meaning of each question and response items.

### Cognitive interviews

A convenient sample of 50 adult PLWH were enrolled in the cross-sectional survey. All participants were recruited through the NGOs supporting PLWH in Alexandria, Egypt during the period from September to November 2023.

After recruitment, participants were divided randomly into five groups (*n* = 10) with an approximate male-to-female ratio of 3:1. A meeting was scheduled with each participant in the first group to fill out the questionnaire and the time needed to complete it was recorded. After completing the questionnaire, each participant was cognitively interviewed face to face using both closed and open-ended questions in a semi-structured interview format aiming to ensure that he/she could understand questions and response items and to determine whether rewording, restructuring, adding, or omitting response items was required. Also, participants were asked if any questions made them feel uncomfortable. After completing the cognitive interviews of the first group of participants, the questions and response items were modified based on the participants’ feedback. Then the process was repeated with the remaining four groups sequentially. Five rounds were sufficient to ensure that the wording of the final version of the questionnaire was clear and participants understood the intended meaning of all questions and response items.

### Statistical analysis

Data were analyzed using IBM’s SPSS Ver.21 (version 21, SPSS, Inc. Chicago, IL, USA). Quantitative data were presented using mean ± SD while qualitative data were presented using frequencies and percentages.

For the cognitive interviews, the total score of each round of interviews was calculated as:

- Percentage of responses that were not identified as having issues with the meaning or construction = (The number of responses for each question of the cognitive interview × 100)/Total answers.

The content validity index (CVI) was calculated for each item (I-CVI) as a proportion of the number of experts in agreement out of the total experts’ opinions for both clearance and relevance, then it was calculated for each section (S-CVI/Ave) as an average of the I-CVI (I-CVI/ number of items in each section) [10].

## Results

### Forward translation and back translation

Two authors translated the original questionnaire from English into Arabic. Minor discrepancies were identified and resolved by both translators and a translated version was synthesized. The translated version was then backward translated by two different independent bilingual translators from Arabic to English where also minor discrepancies were noted and resolved.

### Expert committee review

The expert committee added a question about “the risk factors behind catching HIV infection” to the general information part. On the other hand, the committee made some modifications including omission, addition, or replacement in questions and/or response items related to the COVID-19 pandemic, race, places providing health services for PLWH, supportive services, barriers to receiving services, health insurance services available, and way of covering HIV related medical costs.

After agreement on modifications in questions and response items, the committee made recommendations for the organization of questionnaire sections and their ordering. The approved sequence of sections included starting the questionnaire with the sociodemographic and general data of studied subjects (age, sex, education, years since diagnosis with HIV, place of diagnosis, and risk factors for catching the infection). This is followed by seven sections namely health and medical care, needed services, medical costs and health insurance coverage, HIV medication, HIV-related information, housing, and finally work and income (Table 1).

**Table 1:** Comparison between the original questionnaire and culturally adapted questionnaire

**Modified item**	**Modification**	**Justification of modifications**
Sections’ order	The general information section (age, sex, education, etc.) was ordered first and questions related to HIV- related information were placed as a separate section after the HIV medication section	In the original questionnaire survey report, general information was not reported first After reordering the general information section, questions related to HIV information were more suitable to be placed after the HIV Medication section
Questions related to COVID-19 epidemic	Were omitted from the adapted questionnaire	The original questionnaire was generated in the year 2021 during the COVID-19 pandemic, while translation was carried out in 2023 after the pandemic ended
General information section	- Questions related to race were omitted	- No racial differences are present in Egypt or in Arab countries which are the main target countries/regions for the use of the adapted questionnaire
	- TB clinic and pre-marital examination services center were added to the places of HIV diagnosis	- These are common places for HIV diagnosis as stated by experts and surveyed PLWH
	- A question about the risk factors behind HIV infection was added	- It is an important inquiry as data can be used for planning preventive services towards the most prevalent risk factors in the studied population
Health needs and medical care	- Omitting the following response items: • Trans-gender related services • Language barrier as a cause for not receiving needed care	Not applicable in Egypt and Arab countries
	- Hospital as a place of receiving care was divided into governmental and private hospitals	Based on the healthcare system in Egypt and the Arab countries
	- Treatment history: ▪ Reordering of response items ▪ Addition of chest diseases, sexually transmitted diseases (STDs), and Kaposi sarcoma	- Response items in the original questionnaire were alphabetically ordered while in the translated one they are ordered based on the commonality as stated by experts - Items were added based on experts’ opinions as experienced in their work with PLWH
Needed services (core medical care)	- Addition of TB clinic, dermatological and STD services, obstetrics and gynecology services, and inpatient healthcare	- These items were added based on experts’ opinions as experienced in their work with PLWH
Needed services (supportive services)	- Omitting (food bank, home-delivered meals, and translation services)	- Not applicable/ not commonly encountered in Egypt and Arab countries
Medical costs and health insurance coverage	All questions related to health insurance coverage including types, and sources of payment for health insurance premiums	- These questions should be customized to each country’s system. In the translated questionnaire, they are customized to the health insurance system in Egypt
	Paying costs of HIV medications and HIV-related illnesses	- The response items were adapted to the situation in Egypt where costs are either covered by the national AIDS program, support from non-governmental organizations, or self-paid
Income	Inquiry about receiving financial assistance in the past 6 months was added as a separate question	Based on experts’ opinions as experienced in their work with PLWH

### Content validity

The CVI for all items for relevance and clearance ranged from 0.82 to 1. The CVI for all sections (S-CVI/Ave) for both relevance and clearance respectively were as follows: (0.9,0.85) for health and medical care, (0.94,0.89) for needed services, (0.87,0.92) for medical costs and, health insurance coverage, (0.83,0.85) for HIV medication, (0.97,1) for HIV-related information, (0.96,0.90) for housing, and (0.97,0.86) for work and income. These results denoted adequate content validity (at least 0.82) [10].

### Cognitive interview

The study included 50 PLWH. The age of the studied subjects ranged from 21 to 52 years (the mean = 33.9 ± 8.94 years). About three-quarters (76%) of them were males. The years that passed since diagnosis with HIV ranged from 1 to 6 years (the mean = 2.89 ± 1.8 years). Drug addiction and sharing contaminated syringes were the main risk factors encountered followed by homosexuality and unprotected extramarital sexual relations (Table 2).

**Table 2:** Distribution of studied PLWH based on their sociodemographic characteristics and HIV infection history

**Studied variable**	**Frequency (%)**
Gender	
- Male	38 (76)
- Female	12 (24)
Education	
- Primary school	21(42)
- Middle/high school	14 (28)
- University	15 (30)
Place of diagnosis with HIV	
- NGOs supporting PLWH	23 (46)
- Static/mobile counselling centers	12 (24)
- Primary healthcare centers	11 (22)
- TB clinic	4 (8)
Risk factors for catching HIV	
- Sharing contaminated syringes (drug addiction)	28 (56)
- Homosexuality	13 (26)
- Unprotected extramarital sexual relation	9 (18)

The overall results of the cognitive interview are presented in Table 3. During the cognitive interview process, minor modifications were required based on the participants’ feedback. Modifications included rephrasing some response items as (extramarital sexual relation) was changed to (unprotected sexual relation), (injecting drug abuse) was modified to (sharing contaminated syringes), (visiting outpatient clinic) was replaced with (visiting private clinic), and (Kaposi sarcoma) was changed to (Kaposi sarcoma-AIDS-related cancers). The question covering contacting a physician by phone was modified from “How many times did you need to contact your physician by phone in the past 12 months?” to “Is the service of contacting your treating physician by phone—when needed—available?”. Legal services were added to services needed by PLWH. Moreover, a response item (cannot afford the cost of viral count testing) was added to the response items for the current viral count question.

**Table 3:** Overall results of cognitive interview of the cross-culturally adapted “2021 Louisiana Needs Assessment Questionnaire”

Sequentially interviewed groups	Participant understanding of the intended meaning	The wording was clear for the participant
Group 1	94.7%	85.9%
Group 2	97.5%	89.3%
Group 3	99.2%	93.8%
Group 4	100%	98.6%
Group 5	100%	100%

After the adjustments were carried out following the 4th group cognitive interview, all participants in the last group reported that they understood the intended meaning of questions and found the wording of questions and response items clear. The estimated time needed for completing the self-administered finally tested adapted version was 30 to 40 min (The mean is 33.8 ± 3.31 min).

## Discussion

In the MENA region, Arabic language is the official language in the majority of its countries. So having an Arabic questionnaire is a crucial step in facilitating data collection from their populations. There are a few available translated Arabic questionnaires for PLWH including Health health-related quality of life questionnaire [11], HIV-stigma scale [12], HIV-18 KQ, and social support [13]. There is no valid Arabic questionnaire to assess the unmet health needs of PLWH and related support service needs. The current study followed the published guidelines for the process of cross-cultural adaptation of self-report measures [6] to develop an Arabic culturally-adapted version of the 2021 Louisiana Needs Assessment Questionnaire [9] which was available only in the English language. During the cross-cultural adaptation process modifications were carried out based on suggestions of the expert committee and PLWH who participated in the cognitive interview.

Regarding the questionnaire section's order, the general information was reported towards the end of the original questionnaire survey report. Nevertheless, to build rapport with the participants and ensure adequate participation, sections were reordered based on the expert committee review results. In the adapted questionnaire version, the general information section (age, sex, education, etc.) was ordered first. Also, questions related to HIV-related information were found more suitable to be placed as a separate section after the HIV medication section. Moreover, the re-ordering of response items to questions was done based on the commonality as stated by experts rather than being alphabetically ordered as in the original questionnaire. Rephrasing some questions or response items based on participants’ suggestions as stated in the results was carried out to make it more comfortable, non-offensive, clear, and understandable.

In order to provide critical information for planning and implementing targeted HIV prevention, care, and treatment programs for PLWH and populations at risk [14], a question about “the risk factors behind catching HIV infection” was added to the general information part.

The original questionnaire included questions related to the COVID-19 epidemic as it was generated in the year 2021 during the COVID-19 pandemic. On the other hand, the Arabic translation was carried out in 2023 after the pandemic ended and these questions were no longer relevant. Hence, these questions were omitted from the translated questionnaire. Moreover, questions related to race were omitted as there are no racial differences in Egypt or in the Arab countries, which are the main target countries for the use of the adapted questionnaire.

Many adaptations were carried out to questions related to healthcare services directed to PLWH in order to keep up with the situation in Egypt and Arab countries. Regarding titles of places providing healthcare services for PLWH, they were modified by the expert committee to fit the structure of the healthcare system, and nominations were given to these places in the targeted communities; where “Community health center” and “Community clinic” were replaced by “Governmental health unit”, “Governmental clinics for PLWH care”, “Governmental hospitals” and “Private hospitals”. Also, “TB Clinic” and “Premarital examination services” were added to the place of diagnosis response items. On the other hand, “Being offered a virtual visit” was omitted; and the question related to contacting a physician by phone was rephrased as these services are not available on an ordinary basis. Transgender-related services are also omitted as this is not applicable in Egypt.

For a more comprehensive view of the needed medical services and based on experts’ work experience with PLWH, TB clinic; dermatological and STDs services; obstetrics and gynecology services; and inpatient healthcare services were added to “Needed Medical services” in the adapted questionnaire version. Regarding supportive services, food banks; home-delivered meals; and translation services were omitted because they are not applicable or not commonly provided in Egypt and the Arab countries. On the other hand, legal services were added to the list of services needed by PLWH as suggested by study participants. This may be attributed to that many PLWHs might not find supportive and qualified legal assistance when they face HIV-related discrimination practices in housing, employment, and healthcare services [15, 16].

Moreover, modification of response items related to barriers to receiving different services either medical, social, or psychological support was necessary. “Language barrier” was omitted as the Arabic language is the official language in the Arab countries and is used by the vast majority of the Arab people. “Community conditions and customs preventing me from seeking service” was added to the response items based on participants’ suggestions. This addition reflects self-stigma which may be explained by the results of a previous study conducted to evaluate barriers to adherence to antiretroviral therapy in Egypt where participants experienced stigmatizing practices at treatment facilities and they showed high sensitivity to the behaviors of some healthcare workers [17]. Also, participants suggest adding “the inability to afford viral load testing” as a response item to “the current viral load”. This suggestion could be justified by what was reported by previous studies which found that high prices continue to represent a major barrier to affordable access to both new HIV medicines and viral load testing [18]. In Egypt, although there is an active national AIDS program that provides free access to voluntary testing, counseling, and antiretroviral treatment, it does not provide regular free viral load testing [19].

Regarding medical services funding and health insurance systems, mandatory modifications were carried out to related questions due to the complete difference found between the healthcare system structure and funding in the questionnaire’s place of origin and the situation in Egypt. Egypt's healthcare system is quite diverse and health services in Egypt are provided by three sectors namely government, parastatal, and private sectors. The government sector is funded by the Ministry of Finance. The parastatal sector represents quasi-governmental organizations including the Health Insurance Organization (HIO), the Curative Care Organization (CCO), and the Teaching Hospitals and Institutes Organization (THO). The private sector includes for-profit and non-for-profit organizations [20, 21]. On the other hand, in the place of origin of the original questionnaire, healthcare services are provided and funded through eight health insurance systems as stated in the original questionnaire. Similarly, the response items to “paying costs of HIV medications and HIV related illnesses” were adapted to the situation in Egypt where costs are either covered by the national AIDS program, support from non-governmental organizations, or self-paid.

### Strengths and limitations

Strengths of the current study include providing an Arabic-translated and culturally adapted needs assessment questionnaire for PLWH. The culturally-adapted questionnaire will ease the way to assess the needs of PLWH in Egypt as well as other Arab countries in the MENA region which will facilitate the planning of services and evaluate the impact of the planned services on PLWH to control the growing epidemic in the region. Moreover, the Arabic version could be used in non-Arabic-speaking countries when targeting Arabic-speaking immigrants. Also, the developed questionnaire originated from a regularly used questionnaire for surveying the needs of PLWH in Louisiana by the Department of Health’s Office of Public Health aiming to “provide an estimate of the extent of PLWH’s unmet primary care and HIV-related support service needs, experiences in accessing those services, perceived barriers to those services, and insight into their reported knowledge of those services.” This adds to the questionnaire’s validity. The methodology and the involved sample size are considered adequate according to cross-cultural adaptation guidelines [6].

In the current work, the content validity of the translated version was adequately assessed as well as the face validity using the cognitive interview approach. However, it does not include an assessment of the psychometric properties of the questionnaire that should be covered in future work.

## Conclusion

The synthesized cross-culturally adapted questionnaire is a valid tool to be used among Arabic speakers. It is adapted for use in Egypt. Also, it can be used in other Arabic-speaking populations after minor adaptation related to each country especially in relation to the types of services provided and the adopted health insurance systems. Moreover, it can be used among Arabic-speaking immigrants in foreign countries. Using this questionnaire in surveying the needs of PLWH will provide valid information for planning services to fill their unmet needs. It can also be used for assessing the effectiveness of planned services by comparing the results of surveys conducted before and after the implementation of these services. Further research is recommended to assess the psychometric properties of the developed culturally-adapted questionnaire.

## Supplementary Information

Supplementary Material 1: Appendix 1. Adapted questionnaire

## Data Availability

The datasets used during the current study are available from the corresponding author upon reasonable request. The generated culturally adapted version of the needs assessment questionnaire is included in this published article (Appendix 1).

## Abbreviations

AIDS: Acquired immunodeficiency syndrome
ART: Antiretroviral therapy
CVI: Content Validity Index
HIV: Human immunodeficiency virus
NGOs: Non-governmental organizations
PhD: Doctor of Philosophy
PLWH: People living with HIV
UNAIDS: The Joint United Nations Program on HIV/AIDS

## References

[1] UNAIDS. Miles to go closing gaps breaking barriers righting injustices. Geneva: UNAIDS; 2018. Available from: https://www.unaids.org/en/resources/documents/2018/global-aids-update. Accessed 31 Dec. 2023.

[2] UNAIDS. Global data on HIV epidemiology and response. Geneva: UNAIDS; 2019. Available from: https://www.unaids.org/sites/default/files/media_asset/2019-UNAIDS-data_en.pdf. Accessed 31 Dec., 2023.

[3] Alkaiyat A, Weiss MG. HIV in the Middle East and North Africa: priority, culture, and control. Int J Public Health. 2013;58(6):927–37. 10.1007/s00038-013-0485-y. 23824483 10.1007/s00038-013-0485-y

[4] Abu-Raddad KJ, Akala FA, Semini I, Riedner G, Wilson D, Tawil O. Characterizing the HIV/AIDS epidemic in the Middle East and North Africa: time for strategic action. Washington DC: World Bank; 2010. Available from: http://hdl.handle.net/10986/2457. License: CC BY 3.0 IGO.

[5] Ibrahim K, Rahayuwati L, Herliani YK, Pramukti I. Healthcare needs among people living with HIV: the implication of continuum of care. HIV AIDS (Auckl). 2023;15:235–46. 10.2147/HIV.S403510. 37229313 10.2147/HIV.S403510 PMC10204712

[6] Beaton DE, Bombardier C, Guillemin F, Ferraz MB. Guidelines for the process of cross-cultural adaptation of self-report measures. Spine (Phila Pa 1976). 2000;25(24):3186–91. 10.1097/00007632-200012150-00014. 11124735 10.1097/00007632-200012150-00014

[7] Alaqil AI, Gupta N, Alothman SA, Al-Hazzaa HM, Stamatakis E, del Pozo Cruz B. Arabic translation and cultural adaptation of sedentary behavior, dietary habits, and preclinical mobility limitation questionnaires: a cognitive interview study. PLoS One. 2023;18(6):e0286375. 10.1371/journal.pone.0286375. 37307255 10.1371/journal.pone.0286375 PMC10259774

[8] Guillemin F, Bombardier C, Beaton D. Cross-cultural adaptation of health-related quality of life measures: literature review and proposed guidelines. J Clin Epidemiol. 1993;46:1417–32. 8263569 10.1016/0895-4356(93)90142-n

[9] Louisiana Department of Health, Office of Public Health STD/HIV/Hepatitis program. 2021 Louisiana needs assessment for people living with HIV, Statewide report. 2022. Available from: https://ldh.la.gov/assets/oph/HIVSTD/2021-PLWH-Needs-Assessments/PLWH_Statewide_NeedsAssessmentReport_22Sept26.pdf. Accessed 8 Apr 2024.

[10] Yusoff MSB. ABC of content validation and content validity index calculation. Educ Med J. 2019;11(2):49–54. 10.21315/eimj2019.11.2.6.

[11] World Health Organization. WHOQOL-HIV BREF. Geneva: Department of mental health and substance dependence; 2002. Available from: https://www.who.int/mental_health/media/en/613.pdf.

[12] Aziz MM, Badahdah AM, Mohammed HM. Cross-cultural adaptation and psychometric assessment of an Arabic version of the health care provider HIV/AIDS Stigma Scale. J Int Assoc Provid AIDS Care. 2021;20:1–8. 10.1177/23259582211066402. 10.1177/23259582211066402 PMC8689598 34913384

[13] Terra M, Baklola M, Hasabo EA, Shaheen DG, El-Gilany AH, ARO team of collaborators. Translation, validation and cultural adaptation of the Arabic version of the HIV knowledge questionnaire (HIV-Kq-18). PLoS One. 2023;18(4):e0284542. 10.1371/journal.pone.0284542. 37053199 10.1371/journal.pone.0284542 PMC10101484

[14] Abba OJ, Ibraheem IS, Idoko JA. Prevalence and risk factors for HIV/AIDS among male people in prisons in Jos Prison, Plateau State, Nigeria. Nigerian J Parasitol. 2011;32(2):181–6. https://www.ajol.info/index.php/njpar/article/view/99208.

[15] School of Law of UCLA Williams Institute. The legal needs of people living with HIV evaluating access to justice in Los Angeles. 2015. Available from: https://williamsinstitute.law.ucla.edu/publications/legal-needs-people-living-hiv/. Accessed 8 Apr 2024.

[16] Adekoya P, Lannap F D, Ajonye F A, Amadiegwu, S, Okereke I, Elochukwu C, Et al. Experiences of stigmatization and discrimination in accessing healthcare services among people living with HIV (PLHIV) in Akwa Ibom State, Nigeria. HIV/AIDS (Auckland, N.Z.);16:45–58. 10.2147/HIV.S447551. 10.2147/HIV.S447551 PMC10891273 38406768

[17] Kabbash IA, Zidan OO, Shehata YA. Antiretroviral therapy in Egypt: are there any barriers to medication adherence? Egypt J Community Med. 2019;37(2):58–65. 10.21608/ejcm.2019.30916.

[18] Ondoa P, Kim AA, Boender TS, Zhang G, Kroeze S, Wiener J, et al. Access to HIV viral load testing and antiretroviral therapy switch practices: a multicountry prospective cohort study in Sub-Saharan Africa. AIDS Res Hum Retrovirus. 2020;36(11):918–26. 10.1089/AID.2020.0049. 10.1089/AID.2020.0049 PMC7709893 32722958

[19] World Health Organization. Eastern Mediterranean Region. Egypt, HIV/AIDS Programme. Available from: https://www.emro.who.int/egy/programmes/hiv-aids.html#:~:text=Although%20there%20is%20an%20active,low%20prevalence%20of%20HIV. Accessed 23 Dec. 2023.

[20] WHO-EMRO. Health System Profile Egypt: Regional Health Systems Observatory, EMRO. Cairo: World Health Organization; 2006. p. 1–11.

[21] Gericke CA, Britain K, Elmahdawy M, Elsisi G. Health system in Egypt. In: van Ginneken E, Busse R, editors. Health care systems and policies. Health services research. New York, NY: Springer; 2018. 10.1007/978-1-4614-6419-8_7-2.

