# Supplementary material for: Cross-cultural adaptation and validation of the 2021 Louisiana Needs Assessment Questionnaire for Arabic-speaking people living with HIV
Source: J Egypt Public Health Assoc. 2024 Oct 7;99:25. doi: 10.1186/s42506-024-00171-x (PMC11456554; doi:10.1186/s42506-024-00171-x)
Supplement: Supplementary file 1 — Supplementary Material 1: Appendix 1. Adapted questionnaire [file 42506_2024_171_MOESM1_ESM.docx]

**استمارة تقييم احتياجات المتعايشين مع فيروس نقص المناعة المكتسبة**

**معلومات شخصية**

| 1 | **كم عمرك ؟** ..............................عام | |
| --- | --- | --- |
| 2 | **الجنس** | |
|  | 1. ذكر 2. أنثى 3. مثلي الجنس 4. عابر جنسي 5. أخرى |  |
| 3 | **المؤهل التعليمي** |  |
|  | 1. أمي (تجمع كافة البيانات عن طريق المقابلة) 2. أقرأ وأكتب فقط 3. تعليم ابتدائي 4. تعليم إعدادي / ثانوي 5. تعليم متوسط / فوق المتوسط 6. جامعي 7. ما بعد الجامعي (ماجستير – دكتوراة) |  |
| 4 | **ما مدة تعايشك مع فيروس نقص المناعة المكتسبة (عدد سنوات الإصابة منذ التشخيص):** .........عام | |
| 5 | **أين تم تشخيص إصابتك بفيروس نقص المناعة المكتسبة؟** | |
|  | 1. جمعيات المجتمع المدني (لدعم المتعايشين) 2. وحدات / مراكز طب الأسرة (وحدات الرعاية الأولية) 3. عيادة الأمراض المنقولة جنسيًا 4. مراكز المشورة الثابتة/ المتحركة 5. عيادة الدرن 6. مستشفى / الطوارئ 7. بعيادة طبيب خاص 8. السجن / أثناء المحاكمة 9. بنك الدم أثناء تبرعي بالدم 10. اختبارات ما قبل الزواج 11. معمل تحاليل خاص 12. أخرى: (اذكر)........................................... | |
| 6 | **ما هو عامل الخطورة الذي أدى إلى إصابتك بفيروس نقص المناعة المكتسبة؟** | |
|  | 1. العلاقة الزوجية مع شريك مصاب 2. علاقة جنسية غير محمية 3. تبادل المحاقن الملوثة 4. أثناء نقل الدم لي 5. إصابة عمل بالقطاع الطبي 6. من الأم المصابة أثناء الولادة أو الرضاعة الطبيعية 7. أخرى: (اذكر)...................................... | |

| **الجزء الأول: الصحة والرعاية الطبية**  **في هذا الجزء نسأل عن التاريخ الصحي والعلاجي والرعاية الطبية. إجاباتك الصادقة مهمة فهي تساعدنا لفهم نوعية الخدمات الصحية التي تحتاجها أنت والآخرين.** |
| --- |

| 1 | **كيف تصف صحتك عامةً اليوم؟** | | | | | | | |
| --- | --- | --- | --- | --- | --- | --- | --- | --- |
|  | 1. سيئة جدًا | 1. سيئة | | 1. متوسطة | | 1. جيدة | | 1. جيدة جدًا |
| 2 | **هل احتجت إلى أيًا من الخدمات الطبية الآتية خلال ال12 شهرًا الماضية؟** | | | | | | | |
|  | 1. زيارة لعيادة طبيب 2. خدمات الصحة النفسية 3. خدمات رعاية الأمومة (أمراض النساء والتوليد) 4. خدمات طبية من أجل طفلي | | | | 1. الطوارئ 2. علاج الإدمان 3. الدخول إلى المستشفى (حجز بالمستشفى – جراحة) 4. لم أحتاج إلى أيًا من تلك الخدمات | | | |
| 3 | **خلال ال12 شهرًا الماضية ، كم مرة احتجت إلى زيارة عيادة "فيروس نقص المناعة المكتسبة HIV" أو طبيبك المعالج لأمر طارئ؟** | | | | | | | |
|  | 1. لم أحتاج | | 1. مرة واحدة | | 1. مرتين | | 1. ثلاث مرات أو أكثر | |
| 4 | **هل متاح لك التواصل مع طبيبك المعالج "لأمر خاص بإصابتك بفيروس نقص المناعة المكتسبة" تليفونيًا؟** | | | | | | | |
|  | 1. نعم | | | | 1. لا | | | |
| 5 | **أين عادةً تحصل على الرعاية الطبية عامةً (لأسباب طبية ليس لها علاقة بإصابتك بفيروس نقص المناعة المكتسبة)؟** | | | | | | | |
|  | 1. لا أحصل على رعاية طبية 2. وحدة / مركز صحي حكومي 3. مستوصف / مركز صحي خاص 4. عيادة / مستشفى حكومية لمعالجة المتعايشين مع فيروس نقص المناعة المكتسبة. | | | | 1. عيادة طبيب خاص 2. مستشفى حكومي (عام – تأمين صحي) 3. مستشفى خاص 4. أخرى: (تذكر)................................ | | | |
| 6 | **أين عادةً تحصل على الرعاية الطبية الخاصة بإصابتك بفيروس نقص المناعة المكتسبة؟** | | | | | | | |
|  | 1. لا أحصل على رعاية طبية 2. وحدة / مركز صحي حكومي 3. مستوصف / مركز صحي خاص 4. عيادة/ مستشفى حكومية لمعالجة المتعايشين مع فيروس نقص المناعة المكتسبة. | | | | 1. عيادة طبيب خاص 2. مستشفى حكومي (عام – تأمين صحي) 3. مستشفى خاص 4. أخرى: (تذكر)................................ | | | |
| 7 | **أخر مرة كنت تعاني من مشكلة صحية ولم تحصل على الرعاية الطبية المحتاج لها ، ماذا كان السبب؟** | | | | | | | |
|  | 1. دائمًا أحصل على الرعاية الطبية المحتاج لها 2. لم أعرف إلى أين أتوجه 3. لم أستطع الحصول على موعد من الطبيب 4. لم أستطع الحصول على وسيلة مواصلات لمكان الخدمة الطبية 5. لم أشعر أنني مريض وأحتاج إلى خدمة طبية 6. أخشى الإصابة بفيروس كوفيد-19 | | | | 1. لم أستطع تحمل نفقات الخدمة الطبية 2. كنت مشغول بأمور أخرى لها الأولوية 3. لا أريد أن يعرف أحد بإصابتي بفيروس نقص المناعة المكتسبة 4. لدي إعاقة طبية تعوق تحركي مثال (إعاقة في البصر – السمع – الحركة) 5. ظروف وعادات المجتمع الذي أعيش فيه تمنعني من طلب الخدمة الصحية 6. أخرى: (تذكر)..................................... | | | |
| 8 | **أيًا من تلك المواد تعاطيته خلال ال12 شهرًا الماضية؟ (يمكنك اختيار أكثر من إجابة)** | | | | | | | |
|  | 1. التبغ (سجائر – شيشة أو ما شابه) 2. كحول 3. مواد مخدرة (أذكر النوع).......... | | | | 1. أدوية مسكنة (لم توصف لك من قبل الطبيب) 2. أخرى: (تذكر)........................... 3. لم أتعاطى أيًا من تلك المواد خلال ال12 شهرًا الماضية | | | |
| 9 | **خلال الأسبوعين الماضيين هل شعرت بعدم الرغبة أو عدم الاستمتاع في فعل الأشياء نتيجة تغير حالتك المزاجية؟** | | | | | | | |
|  | 1. نعم | | | | 1. لا | | 1. أحيانًا | |
| 10 | **خلال الأسبوعين الماضيين هل شعرت بالإحباط أو الاكتئاب أو اليأس؟** | | | | | | | |
|  | 1. نعم | | | | 1. لا | | 1. أحيانًا | |
| 11 | **إذا كانت الخدمات الآتية متوفرة لك أيًا منها كنت لتستعمله؟ (يمكنك اختيار أكثر من إجابة)** | | | | | | | |
|  | 1. مجموعات الدعم 2. المشورة 3. الأنشطة الاجتماعية 4. برامج الدعم بقيادة المتعايشين الآخرين | | | | 1. برامج التوظيف 2. التعليم 3. الخدمات القانونية 4. لا أريد استعمال أيًا من تلك الخدمات | | | |

**لدراسة التاريخ العلاجي نريد معرفة هل تم تشخيصك سابقًا بأيًا من الحالات المرضية التالية؟ وفي حالة الإجابة بنعم هل احتجت إلى علاج وهل حصلت عليه خلال ال12 شهرًا الماضية؟**

|  |  |  | | **خلال ال12 شهرًا الماضية** | |
| --- | --- | --- | --- | --- | --- |
|  | **الحالة الطبية** | **هل شخصت بتلك الحالة المرضية سابقًا؟** | | **احتجت علاج لتلك الحالة وحصلت عليه** | **احتجت علاج لتلك الحالة ولم أحصل عليه** |
|  |  | **نعم** | **لا** |  |  |
| 12 | الأمراض الجلدية |  |  |  |  |
| 13 | الأمراض المنقولة جنسيًا مثال الزهري ، السيلان .... |  |  |  |  |
| 14 | الدرن |  |  |  |  |
| 15 | الالتهاب الكبدي الوبائي (ب) |  |  |  |  |
| 16 | الالتهاب الكبدي الوبائي (ج) |  |  |  |  |
| 17 | ساركوما كابوسي (سرطان بالجلد) |  |  |  |  |
| 18 | مرض السكري |  |  |  |  |
| 19 | ارتفاع ضغط الدم |  |  |  |  |
| 20 | التهاب المفاصل |  |  |  |  |
| 21 | أمراض الكبد |  |  |  |  |
| 22 | أمراض الكلى |  |  |  |  |
| 23 | أمراض القلب |  |  |  |  |
| 24 | أمراض الصدر |  |  |  |  |
| 25 | السرطان |  |  |  |  |
| 26 | أرتفاع الدهون بالدم / الكوليسترول |  |  |  |  |

| **27** | **إذا لم تتمكن من الحصول على العلاج في أيًا من الحالات المرضية السابق ذكرها ما هو السبب الأساسي؟ (يمكنك أختيار أكثر من إجابة)** | |
| --- | --- | --- |
|  | 1. لا ينطبق فلقد حصلت على العلاج المطلوب 2. لم أعرف إلى أين أتوجه 3. لم أستطع الحصول على موعد من الطبيب 4. لم أستطع الحصول على وسيلة مواصلات لمكان الخدمة الطبية 5. لم أشعر أنني مريض وأحتاج إلى خدمة طبية 6. أخشى الإصابة بفيروس كوفيد-19 | 1. لم أستطع تحمل نفقات الخدمة الطبية 2. كنت مشغول بأمور أخرى لها الأولوية 3. لا أريد أن يعرف أحد بإصابتي بفيروس نقص المناعة المكتسبة 4. لدي إعاقة طبية تعوق تحركي مثال (إعاقة في البصر – السمع – الحركة) 5. ظروف وعادات المجتمع الذي أعيش فيه تمنعني من طلب الخدمة الصحية 6. أخرى: (تذكر)..................................... |

**هل تم تشخيصك سابقًا بأيًا من حالات اعتلال الصحة النفسية التالية؟ وفي حالة الإجابة بنعم هل احتجت إلى علاج وهل حصلت عليه خلال ال12 شهرًا الماضية؟**

|  |  |  | | **خلال ال12 شهرًا الماضية** | |
| --- | --- | --- | --- | --- | --- |
|  | **الحالة اعتلال الصحة النفسية** | **هل شخصت بتلك الحالة المرضية سابقًا؟** | | **احتجت علاج لتلك الحالة وحصلت عليه** | **احتجت علاج لتلك الحالة ولم أحصل عليه** |
|  |  | **نعم** | **لا** |  |  |
| 28 | القلق أو الهلع |  |  |  |  |
| 29 | اضطراب المزاج ثنائي القطب (Bipolar disorder) |  |  |  |  |
| 30 | الاكتئاب |  |  |  |  |
| 31 | أعراض إعتلال الصحة النفسية بعد الأصابة بفيروس كوفيد (مثال القلق ، الاكتئاب ، الإحساس بالوحدة ...) |  |  |  |  |
| 32 | إدمان أيًا من المواد المخدرة |  |  |  |  |
| 33 | اضطراب ما بعد الصدمة (posttraumatic stress disorder) |  |  |  |  |
| 34 | الفصام (الشيزوفرينيا) |  |  |  |  |
| 35 | أخرى: (تذكر).......... |  |  |  |  |

| **36** | **إذا لم تتمكن من الحصول على العلاج في أيًا من حالات اعتلال الصحة النفسية السابق ذكرها ما هو السبب الأساسي؟ (يمكنك أختيار أكثر من إجابة)** | |
| --- | --- | --- |
|  | 1. لا ينطبق فلقد حصلت على العلاج المطلوب 2. لم أعرف إلى أين أتوجه 3. لم أستطع الحصول على موعد من الطبيب 4. لم أستطع الحصول على وسيلة مواصلات لمكان الخدمة الطبية 5. لم أشعر أنني مريض وأحتاج إلى خدمة طبية 6. أخشى الإصابة بفيروس كوفيد-19 | 1. لم أستطع تحمل نفقات الخدمة الطبية 2. كنت مشغول بأمور أخرى لها الأولوية 3. لا أريد أن يعرف أحد بإصابتي بفيروس نقص المناعة المكتسبة 4. لدي إعاقة طبية تعوق تحركي مثال (إعاقة في البصر – السمع – الحركة) 5. ظروف وعادات المجتمع الذي أعيش فيه تمنعني من طلب الخدمة الصحية 6. الخوف من الوصمة المجتمعية 7. أخرى: (تذكر)..................................... |

| **الجزء الثاني: الخدمات المحتاج إليها**  **في هذا الجزء نسأل عن الخدمات التي قد تكون احتجت إليها خلال ال12 شهر الماضية وإذا ما كنت حصلت عليها.**  **المعلومات التي تمدنا بها تساعدنا على معرفة احتياجاتك والآخرين الذين يعيشون في نفس المجتمع.** |
| --- |

**برجاء إطلاعنا على الخدمات الطبية الأساسية والتى احتجت إليها على مدال ال12 شهرًا الماضية.**

|  | **الخدمة الطبية** | **خلال ال12 شهرًا الماضية** | | |
| --- | --- | --- | --- | --- |
|  |  | **لم احتاج إليها** | **احتجت الخدمة وحصلت عليها** | **احتجت الخدمة**  **ولم أحصل عليها** |
| **1** | **العناية بالأسنان** |  |  |  |
| **2** | **زيارة طبيب الأمراض الجلدية** |  |  |  |
| **3** | **زيارة عيادة الدرن** |  |  |  |
| **4** | **علاج الإدمان** |  |  |  |
| **5** | **كشف العيون / النظر** |  |  |  |
| **6** | **زيارة طبيب الأمراض الباطنة** |  |  |  |
| **7** | **عملية جراحية** |  |  |  |
| **8** | **زيارة طبيب أمراض النساء** |  |  |  |
| **9** | **زيارة طبيب في التخصصات الأخرى** |  |  |  |
| **10** | **زيارة الوحدة الصحية**  **Primary medical care** |  |  |  |
| **11** | **زيارة طبيب الأمراض النفسية / الأخصائي النفسي** |  |  |  |
| **12** | **زيارة أخصائي التغذية** |  |  |  |
| **13** | **الدخول إلى المستشفى**  **Inpatient healthcare** |  |  |  |
| **14** | **خدمة طبية منزلية**  **Home healthcare** |  |  |  |

**برجاء إطلاعنا على الخدمات المساعدة التى احتجت إليها على مدار ال12 شهرًا الماضية.**

|  | **الخدمة المساعدة** | **خلال ال12 شهرًا الماضية** | | |
| --- | --- | --- | --- | --- |
|  |  | **لم احتاج إليها** | **احتجت الخدمة وحصلت عليها** | **احتجت الخدمة**  **ولم أحصل عليها** |
| **15** | **رعاية لأطفالي** |  |  |  |
| **16** | **دعم مادي طارئ** |  |  |  |
| **17** | **خدمة قانونية** |  |  |  |
| **18** | **خدمات منزلية (مساعدة في أعمال المنزل)** |  |  |  |
| **19** | **وسيلة انتقال (مواصلات)** |  |  |  |
| **20** | **خدمات التوظيف** |  |  |  |
| **21** | **دعم اجتماعي (زيارة الأخصائي الاجتماعي – مؤسسة خدمة مجتمعية)** |  |  |  |
| **22** | **تثقيف صحي (معلومات صحية وطبية) / المشورة** |  |  |  |
| **23** | **خدمات تعليمية** |  |  |  |
| **24** | **تنمية مهارات وظيفية** |  |  |  |
| **25** | **خدمات الحقن الأمن** |  |  |  |
| **26** | **المعالجة ببدائل الأفيون** |  |  |  |
| **27** | **المشورة للمواظبة على العلاج** |  |  |  |

**برجاء اطلاعنا عن احتياجاتك للمسكن خلال ال12 شهرًا الماضية.**

|  | **السكن** | **خلال ال12 شهرًا الماضية** | | |
| --- | --- | --- | --- | --- |
|  |  | **لم احتاج إليها** | **احتجت الخدمة وحصلت عليها** | **احتجت الخدمة**  **ولم أحصل عليها** |
| **28** | **سكن طارئ** |  |  |  |
| **29** | **دعم مادي لدفع إيجار المسكن** |  |  |  |
| **30** | **دار إيواء** |  |  |  |
| **31** | **سكن شخصي دائم** |  |  |  |
| **32** | **سكن شخصي مؤقت (فندق – إيجار سكن مؤقت)** |  |  |  |

| **33** | **إذا لم تتمكن من الحصول على أيًا من الخدمات السابق ذكرها (الطبية والمساعدة والسكن) ما هو السبب الأساسي؟ (يمكنك أختيار أكثر من إجابة)** | |
| --- | --- | --- |
|  | 1. لا ينطبق فلقد حصلت على الخدمة المطلوبة 2. لم أعرف إلى أين أتوجه 3. لم أعرف كيفية الحصول على الخدمة 4. لم أستطع الحصول على وسيلة مواصلات لمكان الخدمة | 1. لم أستطع تحمل نفقات الخدمة 2. كنت مشغول بأمور أخرى لها الأولوية 3. لا أريد أن يعرف أحد بإصابتي بفيروس نقص المناعة المكتسبة 4. لدي إعاقة طبية تعوق تحركي مثال (إعاقة في البصر – السمع – الحركة) 5. ظروف وعادات المجتمع تمنعني من طلب الخدمة 6. توجهت لمكان تقديم الخدمة ولم أحصل عليها 7. أخرى: (تذكر)..................................... |

| **الجزء الثالث: تكلفة الخدمة الطبية وخدمات التأمين الصحي** |
| --- |

| **1** | **هل أنت مشترك بخدمة تأمين صحي ؟** | | |
| --- | --- | --- | --- |
|  | 1. نعم | | 1. لا |
| **2** | **هل تستخدم خدمات التأمين الصحي المشترك بها؟** | | |
|  | 1. دائمًا | 1. أحيانًا | 1. لا استخدمها |

**إذا كنت تستخدم خدمات التأمين الصحي المشترك بها انتقل للأسئلة التالية إذا كنت لا تستخدمها انتقل إلى رقم 3**

| **2a** | **ما هو نوع خدمة التأمين الصحي المشترك بها؟ (يمكنك اختيار أكثر من إجابة)** | |
| --- | --- | --- |
|  | 1. التأمين الصحي التابع لعملي الحكومي 2. تأمين صحي تابع لعملي بالقطاع الخاص 3. تأمين صحي تابع للنقابة العمالية المشترك بها 4. اشتراك شخصي بشركة تأمين خاصة | |
| **2b** | **ما هي الخدمات التي تغطيها خدمة التأمين الصحي المشترك بها بالأماكن المتعاقد معها؟** | |
|  | 1. كامل تكلفة الكشف بالعيادات الخارجية 2. جزء من تكلفة الكشف بالعيادات الخارجية 3. كامل تكلفة الفحوصات الطبية (التحاليل والأشعات) 4. جزء من تكلفة الفحوصات الطبية (التحاليل والأشعات) | 1. كامل تكلفة العمليات الجراحية 2. جزء من تكلفة العمليات الجراحية 3. كامل تكلفة الإقامة في المستشفى 4. جزء من تكلفة الإقامة بالمستشفى 5. كامل تكلفة العلاج 6. جزء من تكلفة العلاج 7. خدمات طبية مرتبطة بإصابتك بالفيروس (مثال التحاليل الطبية) |

| **3** | **كيف تغطي تكلفة العلاج الخاص بالفيروس؟** | | | | |
| --- | --- | --- | --- | --- | --- |
|  | 1. أحصل على العلاج بالمجان (تتحمل الدولة تكلفة العلاج) | | 1. أدفع التكلفة بنفسي 2. أتلقى مساعدة من منظمات المجتمع المدني لدعم المتعايشين | | |
| **4** | **كيف تغطي تكلفة الرعاية الطبية المتعلقة بإصابتك بالفيروس؟** | | | | |
|  | 1. عن طريق التأمين الصحي المشترك به. 2. استخدم الخدمات الطبية المدعومة من وزارة الصحة. | | 1. اتلقى دعم مادي من جمعيات المجتمع المدني. 2. أدفع كامل التكلفة بنفسي. | | |
| **5** | **ما مدى الصعوبة التي تواجهها أنت وأسرتك في تغطية تكلفة الرعاية الصحية المتعلقة بإصابتك بالفيروس؟** | | | | |
|  | 1. لا توجد أي صعوبة | 1. قليل من الصعوبة | 1. إلى حد ما | 1. صعبة جدًا | 1. لا نستطيع تحمل التكلفة بتاتًا |

| **الجزء الرابع: العلاج الخاص بفيروس نقص المناعة المكتسبة** |
| --- |

| **1** | **خلال ال12 شهرًا الماضية هل امتنعت عن تعاطي أي جرعة من الأدوية الخاصة بالفيروس الموصوفة لك؟** | | | | | | |
| --- | --- | --- | --- | --- | --- | --- | --- |
|  | 1. نعم | | 1. لا | | | | |
| **2** | **إذا كانت الإجابة نعم، ما هو السبب؟ (يمكنك اختيار أكثر من إجابة)** | | | | | | |
|  | 1. لا تتوفر الأدوية بالصيدلية 2. لم استطع تحمل تكلفة العلاج 3. لم أستطع توفير وسيلة مواصلات للذهاب إلى الصيدلية 4. لم يكن لي مكان سكن ثابت 5. قررت أخذ راحة مؤقتة من تعاطي الأدوية | | 1. تسبب لي الأدوية بعض الأعراض الجانية وتجعلني أشعر بالتعب 2. اعتقدت أنني سليم ولا أحتاج للعلاج 3. لدي مشكلة في تذكر مواعيد أخذ الدواء 4. أخرى: (أذكر)..................................... | | | | |
| **3** | **خلال ال3 أيام الماضية ، كم يوم لم تتعاطى جرعة الأدوية الخاصة بالفيروس الموصوفة لك؟** | | | | | | |
|  | 1. ولا يوم | 1. يوم 1 | 1. يومين | 1. ثلاث أيام | | 1. لا أتذكر | |
| **4** | **ما هو عد الفيروس لديك حاليًا؟** | | | | | | |
|  | 1. غير مرئي | 1. أكثر من 200 copies/ml | 1. لم أحصل على نتيجة التحليل | | 1. لم أقم بإجراء التحليل للتكلفة | | 1. لا أعلم |

| **الجزء الخامس: المعلومات عن الإصابة بفيروس نقص المناعة المكتسبة** |
| --- |

| **1** | **ما هو مصدر معلوماتك عن فيروس نقص المناعة المكتسبة؟ (يمكنك اختيار أكثر من إجابة)** | |
| --- | --- | --- |
|  | 1. الطبيب / التمريض 2. الصيدلي 3. المثقف الصحي 4. مؤسسات الدعم من المجتمع المدني (الجمعيات) 5. الأصدقاء/ الأهل | 1. الزوج/ الزوجة 2. الكتبيات 3. وسائل الإعلام (التليفزيون – الراديو) 4. مواقع الانترنت 5. وسائل التواصل الاجتماعي (الفيس بوك – تويتير ..) 6. أخرى : (أذكر)............ |
| **2** | **هل قام أحد بشرح أيًا من الأمور التالية لك خلال العام الماضي؟ (يمكنك اختيار اكثر من إجابة)** | |
|  | 1. الفيروس غير مرئي 2. من أين أحصل على الواقي الذكري وكيفية استخدامه 3. أهمية الحضور لجميع زيارات الطبيب المحددة لك 4. أهمية الالتزام بأخذ العلاج الموصوف لك | 1. كيف تفصح عن إصابتك بالفيروس 2. كيف تحمي الأخرين من انتقال العدوى لهم 3. الجوانب القانونية المرتبطة بإصابتك بالفيروس 4. لم يشرح لي أحد أيًا من تلك الأمور |

| **الجزء السادس: السكن** |
| --- |

| **1** | **أين تعيش حاليًا؟** | | | | |
| --- | --- | --- | --- | --- | --- |
|  | 1. شقة/ منزل أمتلكه أو تمتلكه أسرتي 2. شقة/ منزل إيجار 3. لدى أحد الأقارب | | 1. لدى أحد الأصدقاء 2. بدار رعاية 3. لا أجد مسكن | | |
| **2** | **أين كنت تعيش منذ 6 أشهر؟** | | | | |
|  | 1. شقة/ منزل أمتلكه أو تمتلكه أسرتي 2. شقة/ منزل إيجار 3. لدى أحد الأقارب | | 1. لدى أحد الأصدقاء 2. بدار رعاية 3. لا أجد مسكن 4. بالسجن | | |
| **3** | **ما عدد الأفراد الذين يعيشون معك بنفس المسكن حاليًا؟** | | | | |
|  | أطفال (السن أقل من 18 عام): ........ | | بالغين (السن 18 عام فأكثر):....... | | |
| **4** | **ما عدد الغرف (بما فيها الصالة) بالمنزل الذي تسكن به حاليًا؟** | | | | |
|  | ............................................ | |  | | |
| **5** | **كم عدد الأماكن التي سكنت بها في ال12 شهرًا الماضية:** | | | | |
|  | ........................................... | | | | |
| **6** | **منذ متى وأنت تعيش في مسكنك الحالي؟** | | | | |
|  | 1. ليس لدي مسكن | 1. أقل من 6 أشهر | | 1. 6 أشهر إلى سنة | 1. أكثر من سنة |
| **7** | **خلال ال12 شهرًا الماضية هل واجهت مشكلة في الحصول على مسكن أو في البقاء بنفس المسكن الذي تعيش به؟** | | | | |
|  | 1. نعم | | | 1. لا | |
| ***إذا كانت إجابتك نعم فأجب عن السؤال التالي أما إذا كانت إجابتك لا فانتقل للسؤال رقم 8** | | | | | |
| **7a** | **ما هي المشكلة التي منعتك من الحصول على سكن أو البقاء بنفس المسكن؟** | | | | |
|  | 1. لم يكن لدي المال الكافي للإيجار 2. لم يكن لدي وسيلة انتقال استخدمها للبحث عن مسكن 3. لدي سجل إجرامي | | | 1. شعرت بالاضطهاد والتمييز 2. أسباب تتعلق بإدماني للمواد المخدرة 3. لدي إعاقة جسدية أو ذهنية 4. سبب أخر: (أذكر)................ | |
| **8** | **خلال ال12 شهرًا الماضية كم ليلة لم تجد بها مكان للمبيت؟** | | | | |
|  | ......................................................... | | | | |
| **9** | **كم تدفع أنت أو أسرتك مقابل إيجار المسكن؟** | | | | |
|  | ...................................................... | | | | |
| **10** | **خلال ال12 شهرًا الماضية هل واجهت مشكلة في دفع إيجار المسكن أو فواتير الخدمات المنزلية (مثال: الكهرباء – الغاز – التليفون ....)؟** | | | | |
|  | 1. نعم | | | 1. لا | |
| **11** | **خلال الثلاث سنوات الماضية هل اضطررت لتغيير المسكن لعدم قدرتك على دفع الإيجار أو فواتير الخدمات؟** | | | | |
|  | 1. نعم | | | 1. لا | |

| **الجزء السابع: العمل والدخل المادي** |
| --- |

| **1** | **ما هو عملك الحالي؟** | | |
| --- | --- | --- | --- |
|  | 1. وظيفة بدوام كامل (30 ساعة عمل أو أكثر / الأسبوع) 2. وظيفة بدوام جزئي (29 ساعة عمل او أقل / الأسبوع) 3. عمل مؤقت 4. عمل خاص بي | | 1. على المعاش 2. لا أعمل / ربة منزل 3. طالب 4. أخرى: (أذكر).............. |
| **2** | **خلال ال12 شهرًا الماضية هل كان هناك سبب يمنعك من العمل؟ (يمكنك اختيار أكثر من إجابة)** | | |
|  | 1. لم يمنعني شيئ عن العمل 2. كنت مريض بسبب إصابتي بفيروس نقص المناعة المكتسبة 3. كنت مريض لسبب أخر غير إصابتي بالفيروس | | 1. تم إنهاء عملي (تسريحي من العمل) 2. حصلت على أجازة من العمل 3. كنت بحاجة لرعاية أطفالي 4. أخرى: (أذكر).................... |
| **3** | **كم بلغ دخل أسرتك خلال الشهر الماضي؟** | | |
|  | ..........................جنيهًا مصريًا | | |
| **4** | **هل حصلت على دعم مادي خلال ال6 شهور الماضية؟** | | |
|  | 1. لم احتاج لدعم | 1. نعم | 1. احتجت لدعم ولم أحصل عليه |
